# Premature and early menopause and neuroimaging biomarkers of neurodegenerative diseases: a systematic review

**DOI:** 10.3389/frdem.2026.1912295

**Published:** 2026-08-12

**Authors:** Emily Gallmetzer, Elijah Mak, Firat Kara, Burcu Zeydan, Ekta Kapoor, Chrisandra L. Shufelt, Walter A. Rocca, Kejal Kantarci

**Affiliations:** 1 Department of Radiology, Mayo Clinic, Rochester, MN, United States; 2 Division of Endocrinology, Department of Internal Medicine, Mayo Clinic, Rochester, MN, United States; 3 Division of General Internal Medicine, Mayo Clinic, Jacksonville, FL, United States; 4 Women's Health Research Center, Mayo Clinic, Rochester, MN, United States; 5 Division of Epidemiology, Department of Quantitative Health Sciences, Mayo Clinic, Rochester, MN, United States; 6 Department of Neurology, Mayo Clinic, Rochester, MN, United States

**Keywords:** Alzheimer’s disease, bilateral oophorectomy, early menopause, estrogen deprivation, neuroimaging, premature menopause, sex differences, women’s brain health

## Abstract

**Background:**

Premature and early menopause involve ovarian hormone deprivation, which may contribute to later-life vulnerability to cognitive decline and neurodegenerative diseases. Multimodal neuroimaging biomarkers provide a means of detecting structural, microstructural, functional, and molecular brain changes before clinical symptoms emerge.

**Methods:**

Following PRISMA guidelines, we conducted a systematic search of PubMed. Study quality was assessed using the Newcastle–Ottawa Scale.

**Results:**

Neuroimaging modalities included *in vivo* structural magnetic resonance imaging (MRI) (*n* = 16), functional MRI (*n* = 2), diffusion tensor imaging (*n* = 4), and positron emission tomography (*n* = 3). Women with premature or early menopause showed regionally specific structural and microstructural brain abnormalities, including lower gray-matter volume or cortical thickness in medial temporal, frontal, and basal forebrain regions; white-matter microstructural abnormalities in the anterior corona radiata, corpus callosum, and fronto-occipital tracts; and reduced posterior hippocampal activation during memory encoding. In one study, earlier age at menopause was associated with higher amyloid-*β* (Aβ) burden in women who underwent premenopausal bilateral oophorectomy (PBO), and elevated tau deposition was reported predominantly among women with higher Aβ levels.

**Conclusion:**

Converging multimodal neuroimaging evidence suggests that premature and early menopause may be associated with Alzheimer’s disease-relevant brain changes. However, findings remain heterogeneous, and longitudinal studies are needed to clarify the temporal relationships between ovarian hormone deprivation and neurodegeneration, and to determine the clinical implication of these findings.

## 1 Introduction: premature and early menopause

Menopause involves the permanent cessation of menstruation resulting from the loss of ovarian follicular function. Spontaneous menopause is typically reported around age 50–51 years in Western populations (Hale et al., 2014). Estimates vary across populations and most women transition between ages 45 and 54 years (World Health Organization, 1996; Rocca et al., 2023). However, a group of women undergo premature or early menopause, thereby exposing the brain to estrogen deprivation at a younger age and for a longer duration than expected. Understanding the implications of earlier hormonal transition on the brain has become an increasingly pertinent question in women’s health research, given growing evidence that it may confer lasting vulnerability to cognitive decline and neurodegenerative disease (Kantarci et al., 2025).

Premature menopause is defined as onset before age 40 years, and early menopause as onset between ages 40 and 44 years (Rocca et al., 2023). Both types of menopause may arise spontaneously or be induced as a result of medical interventions, such as chemotherapy or pelvic radiation, or surgical interventions such as premenopausal bilateral oophorectomy (PBO; also referred to as bilateral salpingo-oophorectomy (BSO), when both fallopian tubes are also removed). PBO may be performed with or without concurrent hysterectomy (Shuster et al., 2010). By contrast, hysterectomy with ovarian conservation is not a cause of ovarian function loss, because it stops menstruation but preserves ovarian endocrine function (Rocca et al., 2023). Throughout this review, we use the terms premature menopause (before age 40) and early menopause (ages 40–44), which may arise spontaneously or be induced by premenopausal bilateral oophorectomy or other medical interventions. Premature menopause is sometimes termed premature ovarian insufficiency; however, we retain the menopause terminology for clarity and consistency, and because the evidence linking early estrogen loss to cognitive decline and dementia derives largely from women who underwent PBO (Rocca et al., 2023; Rocca et al., 2024).

The hallmark consequence of spontaneous menopause is the gradual decline of ovarian estradiol (E2) production, accompanied by a decline of progesterone and androgen signaling. Estradiol is the predominant and most biologically active ovarian estrogen during reproductive life, whereas after spontaneous menopause there is a relatively greater production of estrone (Cui et al., 2013). By contrast, PBO causes abrupt and complete loss of estradiol and all other ovarian hormones. In the brain, estrogen has important antioxidative effects (Moosmann and Behl, 1999) as it contributes to the suppression of immune activation led by astrocytes and microglia, as demonstrated in aged mice (Benedusi et al., 2012). As such, early estrogen loss has been associated with neurobiological changes relevant to neurodegenerative vulnerability including increased oxidative stress, accelerated neuroinflammatory processes, and dysregulated iron homeostasis within the central nervous system (Benedusi et al., 2012; Moosmann and Behl, 1999; Shin et al., 2020). Loss of estrogenic support may increase vulnerability to neuronal injury, gliosis, and accumulation of pathological proteins (Hara et al., 2015).

Because ovarian hormones act on multiple organ systems, their early loss has systemic consequences that extend well beyond reproductive health; as the brain is not isolated from the body, this accelerated somatic aging may itself exacerbate brain aging and contribute to vulnerability to neurodegeneration (Rocca et al., 2018). Together, these mechanisms provide a plausible neurobiological basis underpinning the epidemiological observation that women with premature or early menopause carry a higher risk of cognitive impairment and dementia later in life (Lohse et al., 2021; Rocca et al., 2007).

The neurodegenerative changes may precede onset of clinical symptoms by decades, making them difficult to capture before the clinical threshold is reached (Mak et al., 2017). *In vivo* neuroimaging modalities, including structural MRI, functional MRI (fMRI), diffusion tensor imaging (DTI), and positron emission tomography (PET), can detect early brain changes relevant to neurodegeneration and preclinical Alzheimer’s disease (AD), including gray matter atrophy, white matter microstructural alterations, altered functional connectivity, and amyloid-*β* (Aβ) or tau accumulation before overt clinical symptoms emerge (Mak et al., 2017). These modalities provide complementary measures of preclinical brain changes: structural MRI assesses global and regional gray matter loss, diffusion imaging characterizes white matter microstructural integrity, fMRI evaluates alterations in neural activity and connectivity, and PET detects molecular markers of AD including Aβ and tau deposition in the brain (Brown et al., 2023; Coughlan et al., 2023; Mielke et al., 2024).

Together, these neuroimaging methods provide an opportunity to investigate whether early estrogen deprivation is associated with AD-related and other neurodegenerative brain changes (Mielke et al., 2024). For example, PBO before age 46 has recently been associated with entorhinal cortical thinning and AD imaging biomarkers later in life (Kantarci et al., 2025). These findings suggest that early surgical loss of ovarian function may be linked to later-life vulnerability in AD-sensitive medial temporal regions and AD-related molecular pathology. However, the broader neuroimaging evidence remains heterogeneous across study design, menopause definition, and imaging modality, and it is unclear whether consistent patterns emerge.

The purpose of this systematic review is to summarize the existing literature on premature or early loss of ovarian function, occurring either spontaneously or by surgical or medical interventions, in relation to MRI and PET neuroimaging biomarkers of neurodegeneration or AD. Specifically, we examined the extent to which premature or early loss of ovarian estrogen is associated with changes in brain volume, white matter integrity, and Aβ/tau burden. In addition, we evaluated methodological heterogeneity across studies, such as differences in menopause definitions, imaging approaches, and study design, that may influence the interpretation of findings.

Moreover, because there is considerable variation in the nomenclature across the literature, we use hormone therapy (HT) as an umbrella term throughout. Estrogen replacement therapy (ERT) refers to estrogen-only therapy after PBO with hysterectomy; hormone replacement therapy (HRT) to combined estrogen-plus-progestogen therapy after PBO with uterine conservation; and menopausal hormone therapy (MHT) to hormone therapy in women experiencing spontaneous menopause. Where original studies use different terminology, we retain the source publication’s terms (The North American Menopause Society, 2022).

## 2 Methods

### 2.1 Search strategy

This systematic review was conducted according to the PRISMA 2020 guidelines (Preferred Reporting Items for Systematic Reviews and Meta-Analyses). The screening was performed by two independent reviewers (E.G. and E.M.). A comprehensive literature search was performed on March 25, 2026, in PubMed, with a focus on the effect of early menopause on neuroimaging of neurodegenerative diseases. The search string was: (Menopause OR menopause OR menopausal OR "menopausal transition" OR perimenopause OR "Primary Ovarian Insufficiency" OR "primary ovarian insufficiency" OR "premature ovarian insufficiency" OR "early menopause" OR "premature menopause" OR Oophorectomy OR oophorectomy OR "bilateral oophorectomy" OR "bilateral salpingo-oophorectomy" OR "surgical menopause") AND (Neuroimaging OR neuroimaging OR "Magnetic Resonance Imaging" OR "magnetic resonance imaging" OR MRI OR "Positron-Emission Tomography" OR PET OR "brain MRI" OR "brain PET" OR "functional MRI" OR fMRI OR "single-photon emission computed tomography" OR SPECT OR "brain imaging" OR "brain volume" OR "brain volume" OR "cortical thickness" OR "gray matter" OR "grey matter" OR "white matter" OR "brain atrophy") AND ("Alzheimer Disease" OR "Alzheimer disease" OR "Alzheimer’s disease" OR "AD" OR Dementia OR dementia OR Cognitive Dysfunction OR "cognitive dysfunction" OR "cognitive decline" OR "cognitive impairment" OR cognition OR "brain aging" OR "neurodegenerative disease" OR "Multiple Sclerosis" OR "multiple sclerosis" OR "MS" OR "Parkinson Disease" OR "Parkinson disease" OR "PD"). PubMed (MEDLINE) was used as the single bibliographic database, because it provides close to comprehensive coverage of the clinical neuroimaging and menopause literature relevant to this review. The PubMed search was conducted without a lower date limit (from database inception) through March 25, 2026, the date on which the final search was run. All retrieved records were imported into Covidence systematic review software. Titles and abstracts were screened independently in Covidence by two reviewers. Full-text review and study-level data extraction were performed using structured tables. Extracted information was subsequently reviewed study by study by two reviewers, and discrepancies or interpretive differences were resolved through discussion and consensus.

### 2.2 Eligibility criteria

Studies were included if they met the following four criteria: (1) focused on premature or early menopause; (2) involved neuroimaging modalities; (3) included comparisons of spontaneous premature or early menopause with controls and/or within-group correlation analyses; and (4) were peer-reviewed articles. Studies were excluded if menopause-related variables were only secondary modifiers of another primary exposure, if surgical history was reported without timing or early-menopause/PBO classification, or if they were review articles, animal studies, preprints, or non-peer-reviewed manuscripts.

### 2.3 Quality assessment

We rated the quality of each included study with the Newcastle-Ottawa Scale (NOS). We used the cohort version for the population-based and registry-based studies and the case–control version for the group-comparison studies (Wells et al., 2014). Each study could earn up to nine stars across three areas: how the sample was selected, how comparable the groups were, and how the outcome or exposure was measured. Two of the scale’s items assess follow-up over time and therefore do not apply to studies that included only a single cross-sectional imaging assessment. We interpreted the first as whether enough time had passed between menopause and imaging for brain changes to become detectable, and the second as whether the imaging data were complete. Two reviewers (E. G. and E. M.) rated each study and settled any differences by discussion. We then grouped the scores into good, fair, or poor quality using the cut-offs from the Agency for Healthcare Research and Quality (AHRQ).

## 3 Results

### 3.1 Study selection and characteristics

**Figure 1:**
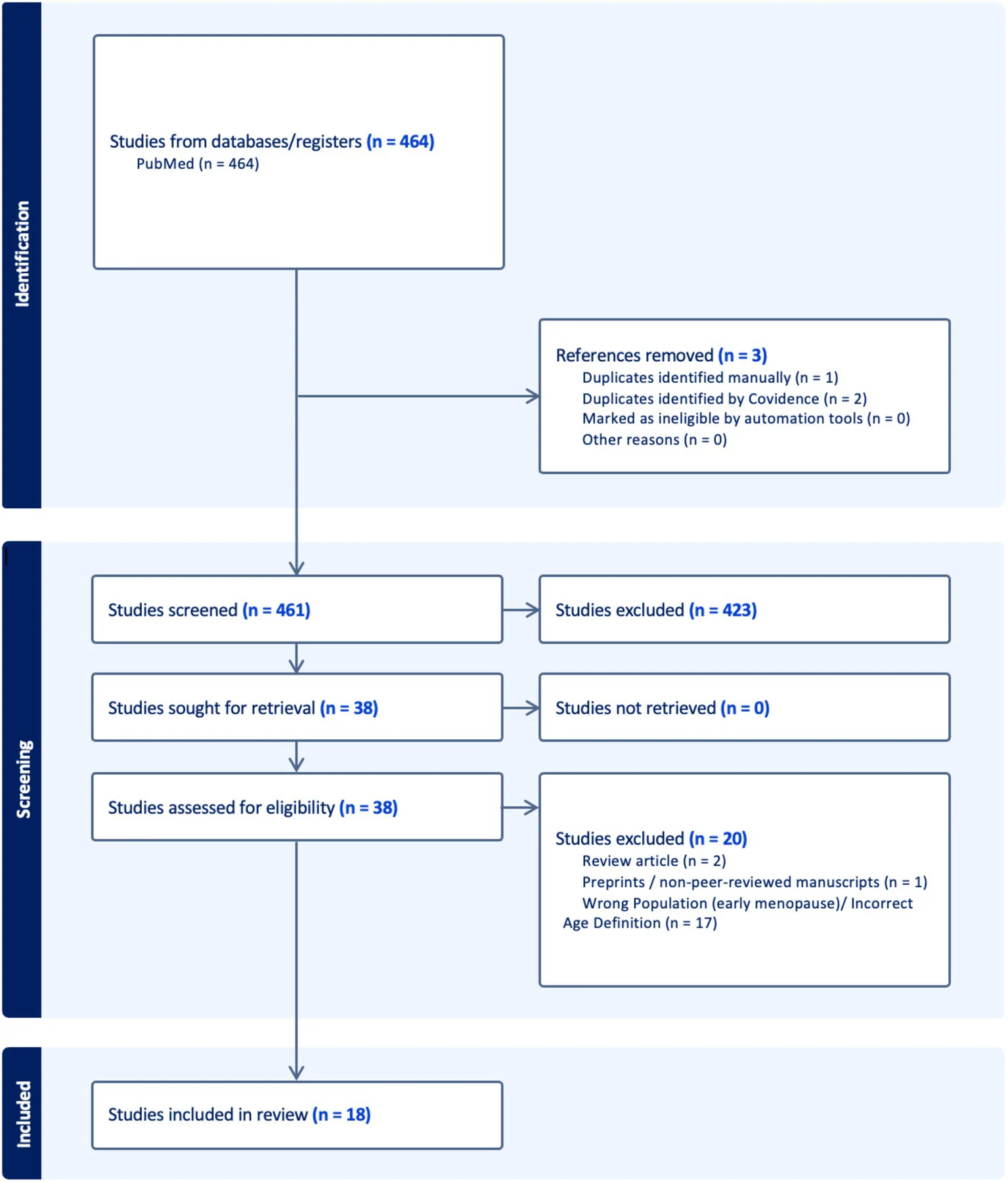
PRISMA flow diagram depicting the study selection process according to PRISMA guidelines. The final review included 18 studies that met all inclusion criteria. PRISMA, Preferred Reporting Items for Systematic Reviews and Meta-Analyses.

A total of 464 records from PubMed were imported into Covidence for screening. Following the removal of 3 duplicates, 461 titles and abstracts were screened by two independent researchers. This led to the exclusion of 423 articles that did not meet the eligibility criteria. Of the remaining 38 full-text articles, 20 were excluded: 15 because of wrong population, exposure, or definition of premature or early menopause; two because menopause-related variables were not the primary exposure of interest; two because they were review articles; and one because it was a preprint. The complete study selection process is illustrated in the PRISMA flow chart (Figure 1).

Because many cohort- or registry-based studies included only a single neuroimaging assessment, we classified study design according to both the sampling framework and the imaging time structure. Studies based on population cohorts or registries with one-time imaging were described as population-based cohort studies with cross-sectional MRI, DTI, or PET assessment. Smaller studies comparing exposure-defined groups at a single imaging visit were described as case–control or group-comparison studies. Of the 18 included studies, 11 were case–control or group-comparison studies, and seven were cohort-based studies with cross-sectional neuroimaging or PET assessment. Definitions of premature or early menopause varied and included PBO/BSO, spontaneous menopause age cutoffs, premature menopause (<40 years), and continuous age-at-menopause analyses. Neuroimaging modalities included structural MRI or MRI-derived structural measures (*n* = 16), functional MRI (*n* = 2), diffusion tensor imaging (*n* = 4), PET imaging (*n* = 3), and cerebrovascular reactivity assessed by transcranial Doppler with MRI-derived brain measures (*n* = 1). Modalities were not mutually exclusive. A detailed summary of the clinical characteristics can be found in Table 1.

**Table 1:** Study characteristics.

Author	Study design	Population (cohort/subcohort *n*)	Participant age at neuroimaging/assessment	Comparator/reference group	Neuroimaging method
Boyle et al. (2021)	Case–control	Cardiovascular Health Study Memory Study; MRI women *n* = 562	79.4 (4.2)	Later menopause comparator; MHT nonusers	sMRI
Brown et al. (2023)	Case–control	Canadian BRCA/BSO cohort; *n* = 72 (AMC *n* = 25, BSO no ERT *n* = 15, BSO + ERT *n* = 16, SM no HT *n* = 16)	33–59; group means: AMC 43.0, BSO 46.5, BSO + ERT 44.8, SM 56.1	AMC with intact ovaries; SM without HT	sMRI; fMRI
Brown et al. (2025a)	Case–control	Canada/Sweden BSO fMRI cohort; *n* = 103 (AMC 40, BSO + ERT 35, BSO no ERT 28)	44.0–45.6 by group	AMC with intact ovaries	sMRI; fMRI
Brown et al. (2025b)	Case–control	Canadian BSO MTL cohort; *n* = 85 (AMC 34, BSO + ERT 28, BSO no ERT 23)	43.8–46.0 by group	AMC with intact ovaries; BSO + ET comparator	sMRI
Calvo et al. (2025)	Case–control	Canadian BRCA cognition cohort; *n* = 333; MRI subset *n* = 177	Age at assessment/imaging: full cohort mean 43.5; group means 38.0–55.0	BRCA controls, AMC, and spontaneous-menopause control	sMRI
Coughlan et al. (2023)	Cohort with cross-sectional PET assessment	Wisconsin Registry for Alzheimer Prevention; PET sample *n* = 292, including females *n* = 193	67 (49–80)	Spontaneous menopause >45; males for sex comparisons	PET (tau and Aβ)
Crestol et al. (2024)	Population-based cohort with cross-sectional MRI/DTI assessment	UK Biobank; MRI sample *n* = 13,780 (premenopause 920, spontaneous postmenopause 12,554, bilateral-oophorectomy 306)	MRI group means 51.3–66.3	Premenopausal and spontaneous postmenopausal women	sMRI; DTI
Gervais et al. (2022)	Case–control	Canadian BRCA/BSO cohort; *n* = 83; volumetric MRI subset *n* = 64	Group means 44.2–56.7	AMC; spontaneous-menopause controls; BSO + ERT comparator	sMRI
Gervais et al. (2023)	Case–control	Canadian BRCA/BSO sleep-MRI cohort; *n* = 62	Group means 43.1–55.9	AMC; peri/spontaneous-menopause controls; BSO + ERT comparator	sMRI
Kantarci et al. (2025)	Population-based cohort with cross-sectional MRI/PET assessment	MOA-2/MCSA/REP; *n* = 231 (early PBO 61, late PBO 51, referents 119)	Median 64–67 by group	Referents without PBO	sMRI; PET
Liao et al. (2023)	Population-based cohort with cross-sectional MRI assessment	UK Biobank; dementia cohort *n* = 154,549; MRI subset *n* = 11,604	Recruitment age 37–73	Menopause ≥ 50	sMRI
Mensegere et al. (2024)	Case–control	TLSA Bangalore; *n* = 528 (early ≤45 *n* = 144; >45 *n* = 384)	Median 61 (>45) and 63 (≤45)	Menopause >45	sMRI
Mielke et al. (2024)	Population-based cohort with cross-sectional DTI assessment	MCSA; DTI sample *n* = 1,011 (PBO < 40 *n* = 22, 40–44 *n* = 43, 45–49 *n* = 39, referents *n* = 907)	Median 72–75 by group	No PBO before 50	DTI
Moir et al. (2023)	Case–control	US CVR MRI study; *n* = 35 (early spontaneous *n* = 19; late spontaneous *n* = 16)	55–68	Late spontaneous menopause ≥ 53	sMRI (brain volume, WMH); cerebrovascular reactivity by transcranial Doppler
Schwarz et al. (2026)	Population-based cohort study with cross-sectional MRI assessment	Cam-CAN; postmenopausal women *n* = 747; structural MRI subset *n* = 182	73.6 (11.6)	No discrete control group; continuous models	sMRI
Witt et al. (2024)	Case–control	Swedish BRCA/BSO pilot; *n* = 39 (BRCA-preBSO 9, BSO no ERT 10, BSO + ERT 10, AMC 10)	Group means 39.2–48.4	AMC; BRCA-preBSO mutation carriers	sMRI
Yuan et al. (2025)	Case–control	Shanghai hospital POI sample; *n* = 84 (POI 33; controls 51)	POI 34.3 (5.4); controls 32.5 (5.8)	Healthy women with regular cycles	sMRI; DTI
Zeydan et al. (2019)	Population-based cohort study with cross-sectional neuroimaging	MOA-2/MCSA imaging sample; *n* = 43 (BSO 23; controls 20)	Median 65 (BSO) and 63 (controls)	No BSO before age 50	sMRI; DTI; PET

AMC, age-matched control; BRCA, breast cancer gene; BSO, bilateral salpingo-oophorectomy; CVR, cerebrovascular reactivity; DTI, diffusion tensor imaging; ERT, estrogen replacement therapy; ET, estradiol therapy; fMRI, functional magnetic resonance imaging; HT, hormone therapy; MHT, menopausal hormone therapy; MRI, magnetic resonance imaging; PBO, premenopausal bilateral oophorectomy; PET, positron emission tomography; POI, premature ovarian insufficiency; SM, spontaneous menopause; sMRI, structural MRI; Aβ, amyloid β; WMH, white matter hyperintensities. For large cohort studies where imaging-age summaries were not separately reported, baseline or recruitment age is given. Values are means unless specified as median, range, or group means. Comparator/reference group column: ‘control’ denotes an unexposed or reference group; ‘comparator’ denotes any other group used for contrast.

### 3.2 Methodological quality (risk of bias)

**Figure 2:**
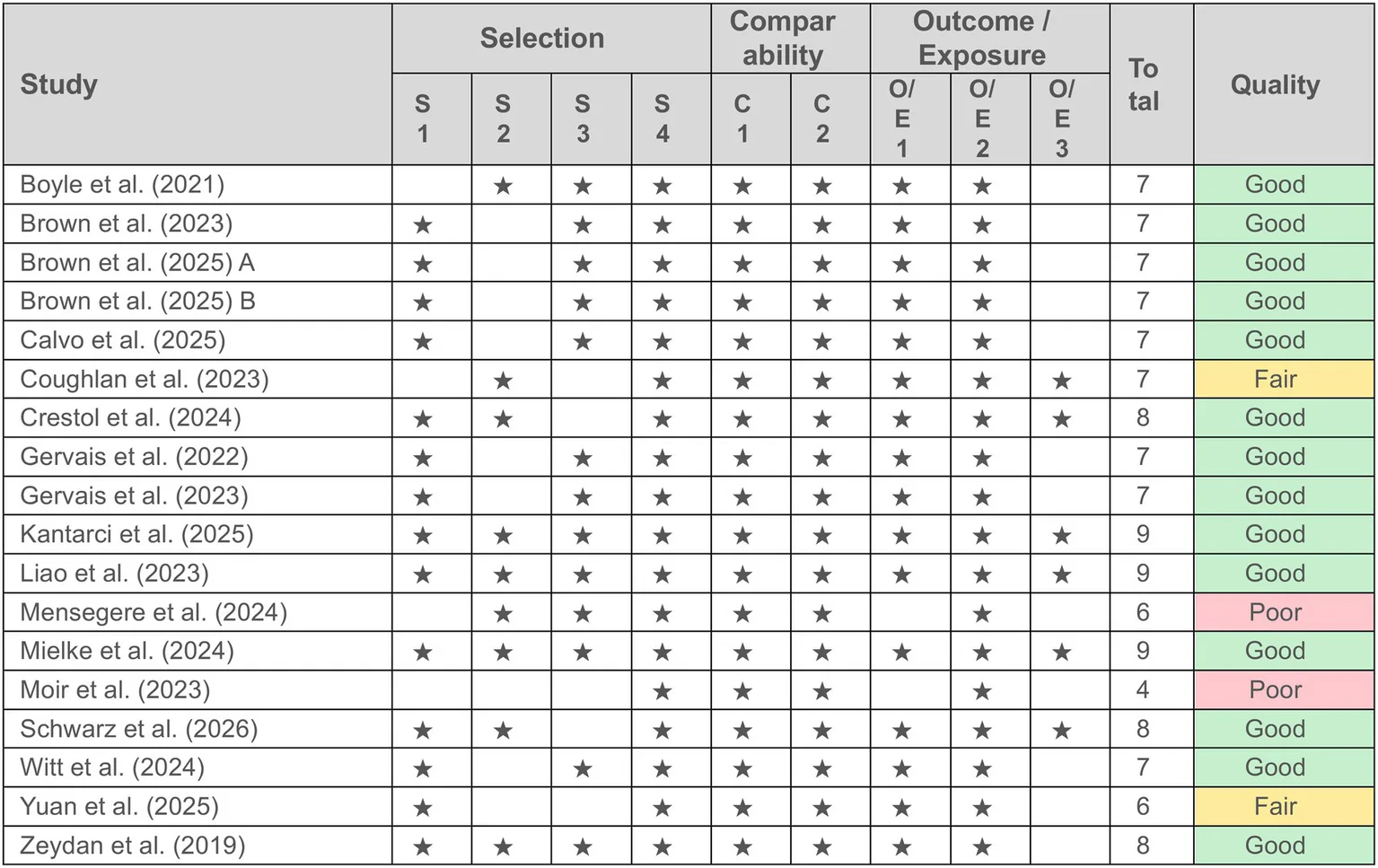
Newcastle-Ottawa Scale (NOS) risk-of-bias assessment of the 18 included studies. A star indicates that the criterion was met (maximum nine stars). Cohort studies were rated with the NOS cohort version and case–control or group-comparison studies with the NOS case–control version. Columns cover the selection (S1–S4), comparability (C1, C2), and outcome or exposure (O/E1–O/E3) domains. Overall quality follows AHRQ standards: Good (green), fair (orange), or poor (red). NOS, Newcastle-Ottawa Scale; AHRQ, Agency for Healthcare Research and Quality.

The quality of the 18 studies was mixed but mostly acceptable (Figure 2). Fourteen studies were rated as good, two as fair (Coughlan et al., 2023; Yuan et al., 2025), and two as poor (Mensegere et al., 2024; Moir et al., 2023). The population-based and registry-based cohorts scored best, because they drew on defined populations, confirmed oophorectomy from medical records, and used objective, automated imaging measures (Crestol et al., 2024; Kantarci et al., 2025; Liao et al., 2023; Zeydan et al., 2019; Mielke et al., 2024). Most case-control and group-comparison studies scored seven stars. They usually lost points because they recruited women from familial cancer clinics rather than the general population, and because they did not report how many women declined to take part (Brown et al., 2023; Brown et al., 2025b; Brown et al., 2025a; Calvo et al., 2025; Gervais et al., 2023; Gervais et al., 2022; Witt et al., 2024). The two poorly rated studies used a single self-reported question to define age at menopause in a small or convenience sample, which weakened their selection and exposure scores (Mensegere et al., 2024; Moir et al., 2023). The most common weaknesses across the studies were the use of self-reported age at menopause in the spontaneous-menopause samples and the fact that almost all studies imaged women only once (Figure 2).

### 3.3 Structural MRI (sMRI)

A total of 16 studies included structural MRI or MRI-derived structural outcomes, including gray-matter volume (GMV), cortical thickness, medial-temporal volumes, white matter hyperintensity (WMH), and brain-age measures. Findings varied across studies but collectively suggested regionally specific rather than global patterns of structural brain differences. Population-based studies using age-at-menopause or menopause-type variables reported mixed but generally regionally specific findings, including lower GMV/WMV and higher WMH in the UK Biobank MRI subset, lower total GMV with earlier menopause in Cam-CAN, and frontal GMV differences in the Tata Longitudinal Study of Aging (TLSA) cohort (Liao et al., 2023; Mensegere et al., 2024; Schwarz et al., 2026).

A recent study showed that earlier spontaneous menopause, defined as menopause at ≤ 49 years, was associated with higher WMH fraction and lower cerebrovascular reactivity compared with late spontaneous menopause, defined as menopause at ≥ 53 years (Moir et al., 2023). In another study, age at menopause, examined both continuously and dichotomized into early versus late menopause, was not independently associated with brain volume (Boyle et al., 2021). However, current and prior MHT use were associated with larger regional brain volumes relevant to cognitive function.

Brain-age findings were limited and mixed. In the UK Biobank study by Crestol et al., no early-menopause-specific brain-age difference was shown for bilateral-oophorectomy menopause, and the bilateral-oophorectomy group had a mean surgery age of approximately 47 years. Because the mean age at oophorectomy in that group was about 47 years, which falls within the usual age at menopause, these results reflect menopause type and brain aging in general rather than premature or early menopause specifically (Crestol et al., 2024).

In women who underwent PBO before age 50 years, smaller amygdala volume and thinner parahippocampal-entorhinal cortex were observed compared with age-matched controls (Zeydan et al., 2019). Similarly, compared with referent women who had not undergone PBO before 50 years of age, women who underwent PBO before age 46 years showed smaller entorhinal cortex thickness independent of age at imaging assessment, whereas lower temporal-lobe cortical thickness was observed only at older ages of imaging assessment (Kantarci et al., 2025).

In women with bilateral salpingo-oophorectomy (BSO) who did not receive any form of HT, smaller regional brain volumes were noted in the dentate gyrus/cornu ammonis 2/3 (DG-CA2/3) hippocampal subfield compared with age-matched premenopausal controls (AMC), whereas the whole hippocampus did not differ between groups (Gervais et al., 2022). In a medial-temporal-lobe volumetric study, BSO without HT was associated with smaller posterior hippocampal and DG-CA2/3 volumes compared with BSO with ET; however, BSO without HT also showed greater perirhinal BA36 volume compared with both BSO with HT and age-matched controls and greater anterolateral entorhinal cortex and perirhinal BA35 volumes compared with controls (Brown et al., 2025a). In an early-BSO cohort, regional brain volumes were not compared between exposure groups; rather, longer sleep latency and lower sleep efficiency were associated with smaller right anterolateral entorhinal cortex volume across the whole cohort (Gervais et al., 2023). Additional evidence suggests that HT may modify BSO-related GMV alterations in a region-specific manner. Compared with age-matched controls, GMV reductions after BSO appeared partially attenuated in some regions among HT users, although the overall pattern was heterogeneous and included localized GMV increases as well as decreases (Witt et al., 2024).

Furthermore, BSO was associated with lower GMV in bilateral frontal regions, including inferior frontal gyrus, precentral gyrus, middle frontal gyrus, supplementary motor cortex, and basal forebrain, compared with BRCA carriers without BSO (Calvo et al., 2025).

In women with idiopathic premature menopause (idiopathic premature ovarian insufficiency), defined as premature menopause without an identifiable surgical, medical, chromosomal, or autoimmune cause, voxel-based analysis showed regional reductions in GMV in the bilateral olfactory cortex and right parahippocampal gyrus, together with lower cortical thickness in bilateral parietal and left insular regions, smaller hippocampal subfield and thalamic subregion volumes, and reduced structural connectivity involving the olfactory cortex, amygdala, hippocampus, and thalamus. No significant global GMV difference was observed in the full sample; however, younger POI participants aged 20-35 showed lower global GMV, cortical thickness, and sulcal depth and higher global gyrification index compared with controls. These subgroup findings were exploratory and uncorrected for multiple comparisons (Yuan et al., 2025).

Overall, structural MRI findings were heterogeneous but suggest regionally specific vulnerability in memory-related, frontal, basal forebrain, and vascular brain systems. However, a universal pattern of global GMV reduction is not supported by the current literature. These inconclusive findings may also be attributed to the heterogeneity in early-menopause definitions, menopause type, exposure timing, and the primary exposure investigated in each study.

### 3.4 Functional MRI (fMRI)

fMRI facilitates the characterization of neuronal activity patterns and connectivity of cognitive networks. This technique was used in two of the studies, both included task-based fMRI investigations in the context of BSO before spontaneous menopause (Brown et al., 2023; Brown et al., 2025b).

Women who underwent BSO and did not receive HT showed lower right posterior lateral hippocampal activation during task-based fMRI for Novel compared with Repeat face-name encoding, compared with women with BSO who received HT (Table 2). Whole-brain analyses revealed a similar activation pattern across novel versus repeated face-name encoding in women with BSO without HT and women in spontaneous menopause, whereas women with BSO receiving HT showed a distinct activation pattern (Brown et al., 2023).

**Table 2:** Menopause definition and key findings.

Author	Definition of early menopause	Comparison with guideline categories	Key findings
Boyle et al. (2021)	Age at menopause (continuous and binary); no cutoff reported; menopause cause not specified	Not comparable; cutoff and menopause cause were not specified	Age at menopause had no independent effect on brain volume Current and prior HT use were associated with larger regional brain volumes in frontal, temporal, and parietal regions In exploratory analyses, bilateral oophorectomy and hysterectomy each showed significant associations with larger parietal volume and lower ventricular volume
Brown et al. (2023)	BSO before spontaneous menopause; no fixed cutoff	Although mean BSO ages align with early/premature categories, no strict age cutoff was used.	BSO without HT showed lower right posterior lateral hippocampal activation during novel face-name encoding than BSO + HT BSO without HT showed lower right posterior lateral hippocampal activity than BSO + HT for the Novel–Repeat face-name encoding contrast. SM showed the same reduced activation pattern as BSO without ERT, despite being ~10 years older BSO + HT showed a distinct whole-brain activation pattern from both BSO and SM No significant behavioral differences in face-name task accuracy between BSO groups and controls BSO and SM showed opposite within-hippocampus connectivity patterns during novel encoding (exploratory, trend-level finding)
Brown et al. (2025a)	BSO before spontaneous menopause; no categorical cutoff	Although mean BSO age was about 40 to 41, no strict age cutoff was used.	Both BSO groups (with and without ET) showed lower posterior hippocampal activation during novel face-name encoding compared to AMC
Brown et al. (2025b)	BSO before spontaneous menopause; mostly before 48, with three participants aged 49–50	Broader cutoff. Although mean BSO ages align with early/premature categories, no strict age cutoff was used.	BSO without ET had smaller posterior hippocampal and DG-CA2/3 volumes than BSO + ET BSO without ET had greater perirhinal BA 36 volume than both BSO + ET and AMC BSO without ET also had greater anterolateral entorhinal cortex and perirhinal BA 35 volumes relative to AMC
Calvo et al. (2025)	BRCA1/2 with BSO; mean BSO age 44; BSO after 49 excluded	Broader cutoff. The <50-year inclusion criterion covers both early menopause (age 40 to 44) and usual-age menopause (age 45 to 49), making the exposure broader than current guideline definitions, although the mean age at BSO falls within the early menopause range. Reported associations not specific to premature or early menopause.	BSO was associated with lower GMV in bilateral frontal regions (inferior frontal gyrus, precentral gyrus, middle frontal gyrus, supplementary motor cortex) and basal forebrain compared with BRCA carriers without BSO
Coughlan et al. (2023)	Premature <40; early 40–45; regular >45	Premature <40 aligns; early 40–45 includes age 45 and cause was not separated	Neocortical Aβ PET × age-at-menopause interactions were observed across multiple regional tau PET measures, with earlier menopause associated with higher tau at higher Aβ levels
Crestol et al. (2024)	No primary definition for early menopause was established, as the main exposure was general menopause type and age. Secondary sensitivity analyses defined premature menopause as 40 years or younger and early menopause as 40 to 45 years.	Not primarily an early-menopause exposure; bilateral-oophorectomy group mean age was about 47	No early-menopause-specific brain-age difference was shown for bilateral-oophorectomy menopause
Gervais et al. (2022)	BSO before spontaneous menopause and at least 1 year before study	Although mean BSO age was about 40 to 41, eligibility was broader than a 45-year cutoff, so no strict early-menopause cutoff was applied.	BSO without estradiol therapy was associated with smaller DG-CA2/3 hippocampal subfield volumes and reduced scene recognition efficiency BSO + ET and spontaneous-menopause groups were unaffected
Gervais et al. (2023)	Ovarian removal before 49 and before spontaneous menopause	Broader cutoff. Broader than current early category because ovarian removal up to 49 was allowed	Regional brain volumes were not compared by group Longer sleep latency and lower sleep efficiency were associated with smaller right anterolateral entorhinal cortex volume across the cohort
Kantarci et al. (2025)	PBO < 46 and PBO 46–49; all before spontaneous menopause	Broader cutoff. <46 is broader than <45; PBO 46–49 is usual-age menopause	PBO < 46 was associated with thinner entorhinal cortex at all ages and with higher Aβ load and temporal lobe atrophy at older ages Tau was elevated only at higher Aβ levels Late PBO showed no differences
Liao et al. (2023)	Menopause age <40, 40–49, and ≥50; MRI used <50 vs. ≥ 50	Broader cutoff. <40 aligns; 40–49 and <50 include usual-age 45–49; the reported associations therefore cannot be attributed specifically to premature or early menopause	Earlier menopause was associated with lower GMV/WMV and higher WMH in the MRI subset
Mensegere et al. (2024)	Early menopause ≤45; comparator >45	≤45 is slightly broader than ≤44; the 45-year group combines premature and early menopause	Menopause ≤45 was associated with lower total gray-matter volume and reduced volumes in left inferior frontal, left middle frontal, and right superior frontal regions (fully adjusted model; left superior frontal did not survive adjustment)
Mielke et al. (2024)	PBO < 40, 40–44, and 45–49	Broader cutoff. Aligned for PBO < 40 and 40–44; 45–49 is usual-age menopause	PBO < 40 was associated with lower FA and higher MD in anterior corona radiata, genu of corpus callosum, and inferior fronto-occipital fasciculus PBO 45–49 had changes in middle frontal and occipital regions PBO 40–44 showed no differences
Moir et al. (2023)	Spontaneous menopause ≤49; late menopause ≥53	Broader cutoff. ≤49 is broader than current early category and includes usual-age 45–49. Reported associations not specific to premature or early menopause	Spontaneous menopause ≤49 was associated with reduced cerebrovascular reactivity and higher WMH compared with late menopause ≥53 Total brain volume did not differ between groups
Schwarz et al. (2026)	Menopause age continuous; descriptive groups <45, 45–55, >55	<45 combines premature and early menopause; cause was not separated	Earlier menopause was associated with lower total GMV White matter volume was not associated with menopause age
Witt et al. (2024)	BRCA-related BSO before age 50 and before spontaneous menopause	Broader cutoff. <50 is broader than current early category and includes 45–49. Reported associations not specific to premature or early menopause	BSO before age 50 was associated with lower GMV in left medial temporal and frontal regions compared with AMC BSO + ERT showed markedly fewer GMV reductions, suggesting partial amelioration by ERT BRCA-preBSO with intact ovaries showed a similar pattern of lower GMV to the BSO group, suggesting an independent effect of the BRCA mutation on brain structure
Yuan et al. (2025)	Idiopathic POI before age 40	Aligned with POI/premature menopause <40; not early menopause 40–44	POI was associated with regional reductions in GMV (bilateral olfactory cortex, right parahippocampal gyrus) and cortical thickness (bilateral parietal and left insular regions), smaller hippocampal subfield and thalamic subregion volumes, and reduced structural connectivity involving the olfactory cortex, amygdala, hippocampus, and thalamus No significant global GMV difference was observed in the full sample (ages 20–40); however, younger POI participants (ages 20–35) showed lower global GMV, cortical thickness, and sulcal depth, and higher global gyrification index compared with controls; no significant global differences were observed in older participants (ages 36–40); findings were exploratory and uncorrected for multiple comparisons
Zeydan et al. (2019)	BSO before age 50 and before spontaneous menopause	Broader cutoff. BSO before age 50, which includes ages 45–49 and is broader than a strict early/premature definition	BSO before age 50 was associated with smaller amygdala volume, thinner parahippocampal-entorhinal cortex, and lower entorhinal white matter FA Hippocampal volume, cortical amyloid on PiB-PET, and WMH volume did not differ between groups

Aβ, amyloid-β; AD, Alzheimer’s disease; AMC, age-matched control; BRCA, breast cancer gene; BSO, bilateral salpingo-oophorectomy; CVR, cerebrovascular reactivity; DG-CA2/3, dentate gyrus/cornu ammonis 2/3; ERT, estrogen replacement therapy; ET, estradiol therapy; FA, fractional anisotropy; GM, gray matter; GMV, gray-matter volume; HT, hormone therapy; MD, mean diffusivity; MHT, menopausal hormone therapy; MRI, magnetic resonance imaging; OR, odds ratio; PBO, premenopausal bilateral oophorectomy; PET, positron emission tomography; POI, premature ovarian insufficiency; SM, spontaneous menopause; TIV, total intracranial volume; WM, white matter; WMH, white-matter hyperintensities; WMV, white-matter volume.

A later study from the same group found lower posterior hippocampal activation during novel face-name encoding in both BSO groups, with and without ET, compared with age-matched controls. Anterior hippocampal activation and raw task accuracy did not differ across groups. This study did not fully replicate the earlier group-specific activation pattern, so these convergent but partly inconsistent results should be interpreted with caution (Brown et al., 2025b).

### 3.5 Diffusion tensor imaging (DTI)

Four studies investigated the microstructural integrity of the white matter through diffusion tensor imaging (DTI). This technique facilitates the quantification of axonal architecture through metrics such as fractional anisotropy (FA) and mean diffusivity (MD). While FA measures the directionality of diffusion, MD quantifies the overall magnitude of water diffusion, serving as an inverse index of membrane density and tissue integrity. Reduced FA, often accompanied by an increased MD, indicates loss of axonal integrity and myelination in the white matter in neurodegenerative diseases.

Women with BSO before age 50 years showed lower entorhinal white matter FA compared with age-matched women without BSO (Zeydan et al., 2019). PBO before the age of 40 years correlated with lower FA and higher MD in the anterior corona radiata, genu of the corpus callosum, and inferior fronto-occipital fasciculus compared with referent women without PBO before 50 years. PBO between ages 40 and 44 years showed no FA or MD difference. By contrast, women with PBO between ages 45 and 49 years showed changes in middle frontal and occipital regions (Mielke et al., 2024).

In spontaneous premature menopause, altered structural connectivity involved the olfactory cortex, amygdala, hippocampus, and thalamus. By contrast, global network topology was relatively preserved (Yuan et al., 2025). In the UK Biobank cohort, Crestol et al. (2024) found no statistically significant evidence implicating PBO in accelerated brain aging (Crestol et al., 2024).

Overall, diffusion findings support alterations in white matter integrity or structural connectivity in some settings of PBO and premature menopause, but the evidence remains limited and heterogeneous.

### 3.6 Positron emission tomography (PET)

PET enables quantitative in vivo assessment of the regional deposition and longitudinal accumulation of AD-related pathologies through the use of radioactive tracers targeting Aβ and tau proteins. Three studies examined the association of Aβ and tau with menopause status.

In cognitively unimpaired women with elevated Aβ, earlier age at menopause was associated with higher regional tau burden, with effects reported in medial temporal, parietal, and temporal-occipital regions. The same study also reported that later HT initiation was associated with higher tau PET across several regions, particularly at higher neocortical Aβ levels (Coughlan et al., 2023).

Women with PBO before age 46 years showed higher Aβ load when imaging was performed at older age, and tau was elevated only at higher Aβ levels. The PBO age 46-49 years group did not show comparable AD-biomarker differences (Kantarci et al., 2025). In Zeydan et al., cortical amyloid on PiB-PET did not differ significantly between BSO and control groups (Zeydan et al., 2019).

Overall, PET evidence remains limited but suggests possible AD-related vulnerability. A clear conclusion cannot be made because further investigations are needed to confirm preliminary findings and because definitions of premature and early menopause differed across studies.

## 4 Discussion

This systematic review summarizes neuroimaging evidence across 18 studies examining the relationship between premature and early menopause and neurodegenerative brain changes. Across structural MRI, diffusion imaging, functional MRI, and PET, findings were heterogeneous but collectively pointed toward regionally specific vulnerability in memory-related and AD-sensitive brain regions. Taken together, the review suggests that earlier ovarian hormone loss may be associated with a pattern of neurodegeneration that overlaps with preclinical AD vulnerability. However, the heterogeneity of study designs and menopause definitions across the literature warrants caution in drawing firm conclusions.

### 4.1 Timing and type of ovarian hormone loss

Across studies, both the timing and the method of ovarian hormone loss emerged as relevant determinants of later brain vulnerability. Studies focusing on surgical menopause via PBO reported more pronounced neuroimaging differences than those examining spontaneous premature or early menopause, which may reflect the more abrupt loss of ovarian hormones following oophorectomy compared with the gradual decline that is typical of spontaneous menopause (Kantarci et al., 2025; Mielke et al., 2024; Zeydan et al., 2019). Collectively, the evidence suggests that earlier and more abrupt ovarian hormone loss is associated with later-life neurobiological vulnerability. However, longitudinal data are needed to confirm the directionality of these associations. The loss of estrogen during a potentially vulnerable period of brain aging may create a distinct state of biological vulnerability, although the evidence remains heterogeneous across spontaneous and surgical menopause studies (Liao et al., 2023).

### 4.2 Structural MRI and brain-age findings

A broad theme emerging from this review is regionally specific vulnerability rather than a diffuse, global pattern of atrophy. Several studies reported differences in medial-temporal regions, including amygdala, parahippocampal-entorhinal cortex, hippocampal subfields, posterior hippocampus, and entorhinal cortex (Brown et al., 2025a; Gervais et al., 2022; Kantarci et al., 2025; Zeydan et al., 2019). Other studies reported lower frontal or basal forebrain GMV, lower total GMV, or higher WMH (Calvo et al., 2025; Liao et al., 2023; Mensegere et al., 2024; Moir et al., 2023; Schwarz et al., 2026). Brain-age findings were limited and mixed, with the only relevant analysis reflecting usual-age surgery rather than early menopause (Crestol et al., 2024). Therefore, current evidence is stronger for regionally specific structural vulnerability than for a diffuse, global brain-age effect.

### 4.3 White matter microstructure and connectivity

Diffusion imaging adds a complementary picture, pointing to compromised white-matter integrity and structural connectivity in some premature menopause and PBO settings. This signal was confined to the youngest age-at-PBO group (before age 40) and absent at ages 40–44 (Mielke et al., 2024), consistent with a threshold or dose–response effect of earlier hormone deprivation, and was accompanied by altered olfactory–limbic–thalamic connectivity in spontaneous premature menopause (Yuan et al., 2025). Notably, the tracts and networks implicated converge on the same frontal and medial-temporal systems that were evidenced by structural imaging, reinforcing a picture of regionally specific rather than diffuse vulnerability. These regions are of interest given their documented involvement in early AD neurodegeneration (Chen et al., 2023), although inference remains hindered by the cross-sectional design of most studies.

### 4.4 Functional MRI

Task-based fMRI, though available in only two studies, provides functional correlates of the structural and microstructural changes described above. Across both studies, reduced posterior hippocampal activation during novel face-name encoding emerged as the most consistent signal (Brown et al., 2023; Brown et al., 2025b), co-localizing with the posterior hippocampal findings from structural imaging and thereby linking altered function to the same medial-temporal substrate. The convergence is only partial, however: the larger study did not replicate the earlier group-specific pattern, and anterior hippocampal activation and task accuracy did not differ across groups. Because both studies originate from the same research group, independent replication in separate cohorts is needed before firm conclusions can be drawn.

### 4.5 PET biomarker findings

PET evidence remains limited but suggests possible AD-related molecular vulnerability, and indicates that a woman’s age at the time of menopause, rather than menopause type alone, is the most sensitive determinant of later molecular vulnerability. Consistent with this pattern, earlier age at menopause was associated with higher regional tau at a given level of Aβ in the WRAP PET cohort, and PBO before age 46 was associated with higher Aβ load at older imaging ages and tau elevation only at higher Aβ levels (Coughlan et al., 2023; Kantarci et al., 2025). Of note, elevated tau deposition was observed predominantly among women with co-occurring elevated Aβ, clustering in MTL, lateral temporal, parietal, and occipito-temporal regions, thus comprising a distribution consistent with Braak staging of early-to-intermediate AD tau pathology (Braak and Braak, 1991; Coughlan et al., 2023; Kantarci et al., 2025). In contrast, Zeydan et al. did not observe significant group differences in cortical amyloid. However, that study examined PBO performed at any age rather than specifically early or premature procedures, which limits direct comparison with the age-at-menopause findings described above (Zeydan et al., 2019). These findings are suggestive but preliminary given the small number of PET studies and differing menopause definitions.

### 4.6 Mechanistic interpretations and clinical implication

Taken together, these findings across imaging modalities point toward a shared underlying mechanism: the early loss of ovarian hormones may weaken the brain’s compensatory reserves and converge on a set of interconnected deleterious molecular pathways. With abrupt and/or early depletion of ovarian hormones, the brain loses its compensatory mechanisms that normally buffer age-related depletion of neurotrophic factors and synaptic plasticity (Brown et al., 2025a; Liao et al., 2023). Several mechanistic pathways may contribute to this association. Reduced estrogen levels may diminish neurotrophic support, leading to loss of synaptic density within the MTL and reduced neuronal resilience to age-related stressors (Brinton, 2008; Hara et al., 2015). Furthermore, estrogen supports cerebral energy metabolism by upregulating glucose transporters. Loss of estrogen may therefore contribute to mitochondrial dysfunction and impaired cellular bioenergetics, resulting in a hypometabolic state where the brain metabolizes its own myelin-derived lipids for energy production (Brinton, 2008). This metabolic shift may increase the production of reactive oxygen species, including superoxide and hydrogen peroxide, which can damage neuronal membranes and further destabilize mitochondrial function (Butterfield and Boyd-Kimball, 2020).

**Figure 3:**
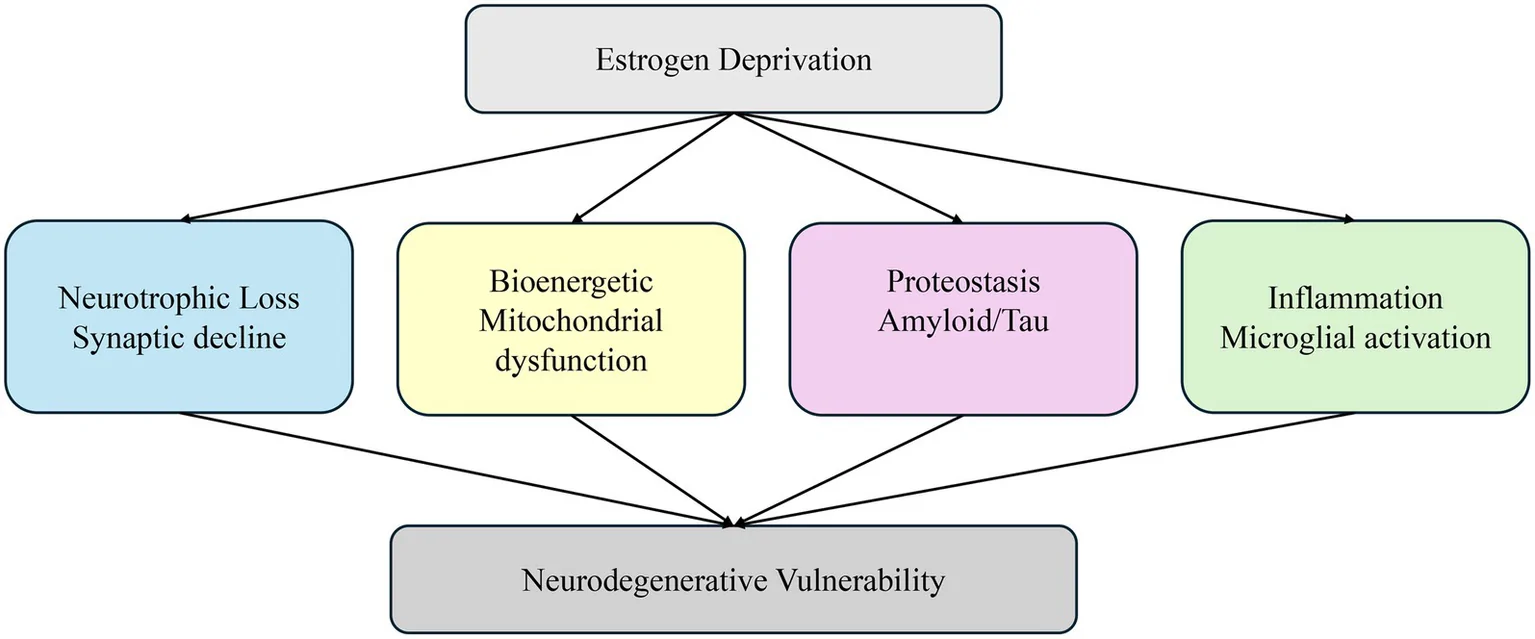
Neurobiological impacts and mechanistic pathways of estrogen depletion.

Additionally, estrogen loss may impair normal protein clearance pathways, as 17-β-estradiol normally regulates enzymes involved in Aβ degradation, such as the insulin-degrading enzyme (Zhao et al., 2011). Reduced estrogen signaling may therefore lead to a failure in the proteolytic clearance of Aβ peptides. Estrogen also supports autophagic flux, and disruption of this process may contribute to intracellular accumulation of misfolded proteins such as hyperphosphorylated tau. Finally, estrogen has important anti-inflammatory effects that help maintain microglia in a homeostatic state. Estrogen depletion may shift the brain toward a pro-inflammatory state, contributing to chronic neuroinflammation (Bustamante-Barrientos et al., 2021; Figure 3). Collectively, these findings underscore the importance of considering reproductive parameters such as timing of menopause onset and type of menopause in clinical risk stratification for AD.

These findings also have wider implications for how premature and early menopause are viewed as dementia risk factors. Premature and early menopause, whether spontaneous or surgical, affect a substantial minority of women and can be identified decades before any cognitive symptoms arise (Rocca et al., 2023). This creates an early window in which reproductive history could help flag women who may benefit from closer monitoring and from timely attention to modifiable co-risk factors such as vascular health. From a public-health perspective, incorporating age at menopause and type of menopause into dementia risk models could improve the identification of at-risk women and inform decisions about the timing of hormone therapy and other preventive measures. Because the current evidence is heterogeneous and largely cross-sectional, these implications are best seen as a rationale for prevention-oriented research rather than as established clinical guidance.

### 4.7 Limitations

There are several limitations of the current research evidence which must be acknowledged. First, most included studies used cross-sectional neuroimaging assessments, whether within case–control/group-comparison designs or cohort-based samples, limiting causal inference about the temporal sequence between ovarian hormone loss and neurobiological change. Consequently, it remains unclear whether the observed changes are direct consequences of hormonal deprivation or whether they are modified by preexisting factors such as cerebrovascular risk factors. The search was also restricted to a single bibliographic database, although PubMed provides broad coverage of the relevant neuroimaging and menopause literature and the reference lists of included studies and prior reviews were screened, a single-database strategy cannot fully exclude publication or indexing bias. Furthermore, the substantial methodological heterogeneity regarding the clinical definition of premature and early menopause complicates a coherent interpretation of different study populations. In particular, several studies used age cutoffs of <45, <46, or ≤49 years, which combine the current premature and early menopause categories with parts of the usual-age range (45–54 years), precluding direct comparison across studies. The definitions of menopause used in the studies and a comparison to our categories can be found in Table 2.

Across the 18 studies, exposure definitions often reached into the usual-age menopause range. Sixteen of the 18 included women whose menopause or oophorectomy occurred at age 45 or older, or set no upper age limit, and only Schwarz et al. (2026) and Yuan et al. (2025) kept the exposure below age 45. Many of the reported associations therefore cannot be tied specifically to premature or early menopause. This is clearest for Liao et al. (2023), who compared menopause before age 50 with menopause at 50 or older. Such heterogeneity limits how specifically the review’s conclusions can be read.

Large studies often rely on volunteer cohorts such as the UK Biobank, and participants who sign up for such studies tend to be healthier than the general population. This healthy-participant bias can modify or obscure meaningful associations and limits generalizability to women in the general population (Crestol et al., 2024; Liao et al., 2023).

The cutoff point for age at menopause has changed over time. Under 46 years was the commonly used cutoff in the past, whereas today premature menopause is defined as occurring before 40 years and early menopause between 40 and 44 years. Applying these definitions consistently would make future studies much easier to compare with one another (Rocca et al., 2023). We emphasize that what drives the observed differences is how old a woman was when she reached menopause and how old she is at the time of imaging testing, not how many years have passed since menopause, because that interval only matters insofar as it tells us something about her current age.

Additionally, differences in neuroimaging modalities such as varying MRI field strength and different image processing algorithms for volumetry pose an additional challenge. Future multi-site studies could harness harmonization approaches of imaging outcomes (Mak et al., 2026). This methodological variability is further compounded by inconsistent or absent reporting of standardized effect sizes, which precludes a clear comparison of results across studies.

### 4.8 Gaps and future directions

Looking forward, large-scale longitudinal studies are warranted to determine the chronological sequence of neurobiological changes during the endocrine transition of spontaneous or surgical menopause. For example, such a study would entail tracking the same women with serial neuroimaging and cognitive assessments over years. This would help determine when brain alterations begin in relation to the drop in estrogen levels. This would also help us to understand how these hormonal changes interact with other individual risk factors. The synergistic interaction between reproductive timing and genetic predisposition, such as the apolipoprotein E ε4 (APOE-ε4) genotype, warrants further evaluation. Whether specific critical windows exist during which hormonal interventions can decelerate the described degeneration processes in networks vulnerable to AD remains to be elucidated. Future research should integrate multimodal imaging to better understand the connection between microstructural damage and the accumulation of pathological proteins.

Other reproductive characteristics, such as parity, may further modify these associations and warrant evaluation as potential effect modifiers, and emerging molecular imaging of neuroinflammation could help clarify the inflammatory contribution to these brain changes. Finally, even though neuroimaging is particularly suited to research settings, emerging blood-based biomarkers may ultimately provide a less invasive and less costly option for risk assessment in clinical practice.

## 5 Conclusion

In conclusion, the findings of this review support the hypothesis that menopause timing and abrupt ovarian hormone loss are relevant to later-life vulnerability to neurodegenerative changes. Although findings were heterogeneous, a convergent pattern of regionally specific vulnerability emerged in memory-related, vascular, and AD-vulnerable systems, spanning gray-matter atrophy, white-matter microstructural deficits, reduced hippocampal activation, and preliminary evidence of altered Aβ and tau burden.

Collectively, these data suggest that women's reproductive history should be considered an integral part of disease risk profiles. The consideration of endocrine factors could therefore pave the way for sex-informed subclinical and clinical pathways that support earlier diagnosis and more precise prevention. Ultimately, incorporating hormonal history into clinical evaluation may improve the development of personalized strategies aimed to preserve cognitive function and reduce the global burden of neurodegenerative diseases.

## Data Availability

The original contributions presented in the study are included in the article, further inquiries can be directed to the corresponding author.
